# The Importance of Intra-aortic Pulse Pressure After Anterior ST-segment Elevation Myocardial Infarction

**DOI:** 10.21470/1678-9741-2018-0106

**Published:** 2018

**Authors:** Ilker Gul, Levent Cerit, Bihter Senturk, Mustafa Beyazıt Alkan, Hatice Kemal, Zeynep Cerit, Belma Yaman, Songul Usalp, Hamza Duygu

**Affiliations:** 1 Department of Cardiology, Near East University Faculty of Medicine, Nicosia, Cyprus.; 2 Department of Cardiology, Dokuz Eylül University Faculty of Medicine, Izmir, Turkey.; 3 Department of Cardiology, Kas State Hospital, Antalya, Turkey.; 4 Department of Pediatric Cardiology, Near East University Faculty of Medicine, Nicosia, Cyprus.

**Keywords:** Myocardial Infarction, Treatment Outcome, Cardiac Catheterization, ST Elevation Myocardial Infarction

## Abstract

**Objective:**

To evaluate the association of pulse pressure (PP) with mortality and major adverse cardiac events (MACE) in one-year period after anterior ST-elevation myocardial infarction (A-STEMI).

**Methods:**

A total of 261 consecutive patients whose blood pressure was measured with the aid of a catheter before primary percutaneous coronary intervention (PPCI) between August 2016 and February 2017 were included in the study. The patients were divided into three groups according to pulse pressure (PP) (Group 1, PP<35 mmHg; Group 2, 35≤PP≤50 mmHg; Group 3, PP>50 mmHg).

**Results:**

The mean age of the patients was 63.4±14.1 years, and 206 of them were male. The groups were similar in terms of age and diastolic blood pressure (DBP). The ratio of female patients in Group 1 was higher, and their systolic blood pressure (SBP) was lower than those from the other groups (*P*=0.005 *vs*. *P*=0.042). The rates of MACE and mortality were higher in Group 1. The predictive PP values were calculated to be 42.5 mmHg for development of MACE and 41.5 mmHg for mortality. One-year survival ratio was worse in Group 1 than in the others according to Kaplan-Meier analysis (*P*<0.001).

**Conclusion:**

The values of PP which was measured intra-aortically in patients with A-STEMI were associated with mortality and MACE in the one-year follow-up period.

**Table:** 

Abbreviations, acronyms & symbols				
**ACS**	**= Acute coronary syndromes**		**LVEDP**	**= Left ventricular end-diastolic pressure**
**AMI**	**= Acute myocardial infarction**		**LVEF**	**= Left ventricular ejection fraction**
**ANOVA**	**= Analysis of variance**		**MACE**	**= Major adverse cardiac events**
**AUC**	**= Area under the curve**		**MRI**	**= Magnetic resonance imaging**
**A-STEMI**	**= Anterior ST-elevation myocardial infarction**		**PAD**	**= Diastolic arterial pressure**
**c-TnI**	**= Cardiac troponin-I**		**PAS**	**= Systolic arterial pressure**
**CI**	**= Confidence interval**		**PP**	**= Pulse pressure**
**CK-MB**	**= Creatinine kinase-myocardial band**		**PPCI**	**= Primary percutaneous coronary intervention**
**CO**	**= Cardiac output**		**RAAS**	**= Renin-angiotensin-aldosterone system**
**CT**	**= Computed tomography**		**Re-MI**	**= Re-myocardial infarction**
**DBP**	**= Diastolic blood pressure**		**ROC**	**= Receiver operating characteristic**
**DtB**	**= Door-to-balloon**		**SBP**	**= Systolic blood pressure**
**ECG**	**= Electrocardiogram**		**SD**	**= Standard deviation**
**GFR**	**= Glomerular filtration rate**		**StD**	**= Symptom-to-door**
**Gp**	**= Glycoprotein**		**STEMI**	**= ST-elevation myocardial infarction**
**HR**	**= Hazard ratio**		**STXs**	**= SYNTAX score**
**IABP**	**= Intra-aortic balloon pump**		**TIMI**	**= Thrombolysis in myocardial infarction**
**IV**	**= Intravenous**			

## INTRODUCTION

Acute myocardial infarction (AMI) is one of the leading causes of mortality worldwide^[1]^. The most important cause of this condition is the decline of cardiac functions after AMI. This decline's severity is associated with the localization and size of the infarct region^[2]^. The inadequacy of cardiac functions causes acute changes in hemodynamic parameters, and eventually, cardiac output (CO) decreases. The neuroendocrine system is activated in response to an impaired CO. The secretory rhythm of vasopressor substances such as noradrenaline and angiotensinogen-II into the circulatory system changes. With the effect of these vasopressors, changes occur in SBP and DBP compared to their baseline values^[3]^.

The changes in blood pressure after acute coronary syndromes (ACS) were demonstrated to be associated with increased morbidity and mortality^[4]^. For this reason, blood pressure levels in risk scoring systems, which are used after ACS, rank as important prognostic indicators^[5]^. Pulse pressure (PP) is the difference between SBP and DBP. PP provides important information about the status of the arterial circulation such as cardiac contraction and stiffness. It is suggested that the power of PP for predicting cardiovascular events is higher than that of SBP and DBP^[6]^. The PP of AMI patients, which is measured with the aid of a cuff from the arm at the time of the first admission to the emergency service, is one of the determinants of in-hospital and post-discharge long-term prognoses^[7,8]^. However, the central blood pressure measured from the aorta has a stronger association with early and long-term outcomes of cardiovascular pathologies than the blood pressure measured from the arm^[9,10]^.

In our study, we measured intra-aortic blood pressures of patients in whom primary percutaneous coronary intervention (PPCI) was planned due to anterior ST-elevation myocardial infarction (A-STEMI) with the aid of a catheter before PPCI. In line with the guidelines, we included patients who had normal blood pressure range. We aimed to evaluate the association of PP with mortality and major adverse cardiac events (MACE) in one-year period after A-STEMI.

## METHODS

The patients of this study were gathered at a tertiary centre between August 2016 and February 2017. The approval from the ethics committee was obtained prior to the study, and all researchers completed the study in accordance with the Helsinki Declaration.

Patients in whom less than 12 hours has passed after the onset of ST-elevation myocardial infarction (STEMI) and who were taken to the angiography laboratory for PPCI were evaluated. Those who had single vessel disease, age between 18 and 80 years, and left ventricular ejection fraction (LVEF) more than 25% were included in our study. Since hypotensive periods were more frequent in inferior STEMIs, creating a homogeneous group was aimed. Therefore, only patients with A-STEMI were included in the study. Electrocardiographic examinations were performed during the period when the patients were first admitted to the emergency department. Presence of ST elevation ≥ 0.2 m in at least two of the precordial leads, newly developed left bundle branch block, presence of chest pain with the typical onset and spread, and cardiac troponin-I (c-TnI) and creatinine kinase-myocardial band (CK-MB) values greater than the 99^th^ percentile were considered as A-STEMI. Since our centre has a team on call for 24/7, the patients were quickly taken to the angiography laboratory after being diagnosed with A-STEMI.

Antiaggregant therapy of these patients (clopidogrel, 600 mg as a loading dose, 75 mg/day as maintenance dose; acetylsalicylic acid, 300 mg as a loading dose, 100 mg/day as maintenance dose) was initiated in the emergency service. Also, concurrently, unfractionated heparin was administered via intravenous (IV) route at the dose of 100 IU/kg. An additional dose of IV unfractionated heparin was administered as needed so that the activated coagulation time would be >250. Beta-blockers, statins, and renin-angiotensin-aldosterone system (RAAS) blockers were initiated after PPCI according to the hemodynamic state in the intensive care unit.

The exclusion criteria included passing more the 12 hours after the onset of A-STEMI, previous AMI history, presence of known heart failure, presence of moderate to high heart valve insufficiency and/or stenosis, glomerular filtration rate (GFR) less than 30 ml/min/1.73 m^2^, chronic pulmonary disease, chronic liver disease, and presence of previous cerebrovascular disease. In addition to these, patients with previously diagnosed hypertension and who were on antihypertensive medication were not included in the study. To minimise the effects of SBP and DBP levels on the study's outcome, the condition of having these values within normal limits (100-140 mmHg for SBP and 60-90 mmHg for DBP) according to current hypertension guidelines was applied. In our study, patients with hemodynamically unstable requirement of inotropic agents and intra-aortic balloon pump (IABP) were excluded.

Blood pressure measurements were performed using a 6F pigtail catheter which was placed in the ascending aorta just before coronary angiography. Before each measurement, the pressure monitor was calibrated to zero. The system was rinsed with heparinised liquid, and it was paid attention not to leave any air inside. The transducer of the system to measure blood pressure was set at the same level with the patient's heart. PP was calculated by subtracting DBP from SBP. The patients were divided into three groups according to their PP: Group 1 included patients with PP <35 mmHg, Group 2 included patients with 35≤PP≤50 mmHg, and Group 3 included patients with PP> 50 mmHg.

The study's endpoints were accepted as all-cause mortality and MACE (death, cardiogenic shock after PPCI, recurrent myocardial ischemia, re-myocardial infarction [Re-MI], and stroke). Cardiogenic shock was defined as persistent hypotension which does not respond to IABP placement, inotropic support, and fluid support after PPCI and circulatory impairment. Re-MI was defined as recurring chest pain, new electrocardiogram (ECG) changes, and increase of cardiac enzymes compared to previous values. Stroke was defined as the presence of focal neurological deficit which persists more than 24 hours and its confirmation with methods such as computed tomography (CT) and magnetic resonance imaging (MRI). The SYNTAX score (STXs) was calculated to determine the prevalence of coronary artery disease (www.syntaxscore.com).

Echocardiographic examinations are performed routinely on AMI patients in the emergency room of our centre; the patients are evaluated quickly, and images are recorded. These examinations can be completed without a significant delay owing to the presence of the angiography team and a cardiologist on call for 24/7 in the emergency. Cardiac function, valve function, and complications at admission were evaluated with echocardiography. The LVEF of all patients was calculated by the modified Simpson method. Patients were followed-up for one year after A-STEMI; they were called to outpatient clinics at 1, 3, 6, and 12 months.

### Statistical Analysis

In our study, the SPSS 17.0 (Chicago, Illinois, USA) program was used for statistical analysis. Data distribution was checked by the Kolmogorov-Smirnov test. Continuous variables were expressed as mean standard deviation (SD) or median (interquartile range), according to their distribution status. Continuous variables with normal distribution were compared with the analysis of variance (ANOVA), those with non-normal distribution were compared with the Wilcoxon rank test. Categorical variables were assessed with the chi-square test. Cumulative survival and MACE curves were plotted, and log-rank analysis was performed using the Kaplan-Meier method. Correlation analyses were performed to determine the relationship between PP and LVEF, c-TnI, CK-MB, and symptom-to-door (StD) time. Univariate analyses were performed with the variables, such as age, gender, diabetes mellitus, heart rate, smoking, hyperlipidemia, SBP, DBP, PP, STXs, LVEF, cTn-I, StD, door-to-balloon time (DtB), GFR, thrombolysis in myocardial infarction (TIMI) flow grade, use of glycoprotein (GP) inhibitor, beta-blockers, and RAAS inhibitors in order to determine the predictors of MACE. Backward stepwise multivariate regression analysis was performed with variables of the female gender, SBP, PP, LVEF, GFR, beta-blockers, RAAS inhibitors, and StD; the *P* values of which was found to be *P*<0.10 were evaluated by univariate analyses. Receiver operating characteristic (ROC) analyses were performed to determine the PP values associated with mortality and MACE. The area under the curve (AUC), cut-off value, sensitivity, specificity, and confidence interval (CI) were determined by ROC analyses. The *P* values <0.05 were accepted as statistically significant.

## RESULTS

Of 297 patients who underwent PPCI with the diagnosis of A-STEMI in our clinic, 261 completed a one-year follow-up. Thirty-six patients who could not come to their control meetings for the study or who could not be contacted afterwards were not included in the statistical analysis. Of the studied patients, 206 were male and 55 were female. The male patient ratio was lower in Group 1. Mean age was calculated as 63.4±14.1 years. Means of smoking patients and SBP were higher in Groups 2 and 3. General characteristics, laboratory data, and medical treatments of the study groups are shown in Table 1.

**Table 1:** Patients’ demographic, laboratory, and angiographic characteristics.

Variable	Group 1 (n=74)	Group 2 (n=107)	Group 3 (n=80)	*P* value
Age (years)	62.7 (± 10.8)	62.8 (±13.4)	65.2 (±14.1)	0.450
Male [n (%)]	50 (67.6%)	87 (81.3%)	69 (86.3%)	0.013*
Female [n (%)]	24 (32.4%)	20 (18.7%)	11 (13.8%)	0.005*
BMI (kg/m^2^)	27.3 (±7.1)	27.7 (±6.9)	27.8 (± 8.1)	0.645
Diabetes mellitus [n (%)]	11 (14.9%)	22 (20.6%)	13 (16.3%)	0.569
Smoking [n (%)]	23 (31.1%)	57 (53.3%)	42 (52.5%)	0.006*
Heart rate (bpm)	82.3 (±13.5)	86.6 (±16.2)	86.8 (±15.1)	0.241
SBP (mmHg)	112.3 (±14.4)	131.2 (± 8.3)	135.2 (± 4.6)	<0.001*
DBP (mmHg)	76.1 (±12.5)	78.7 (±13.7)	74.2 (±9.7)	0.461
Haemoglobin (g/dl)	13.6 (±1.8)	14.1 (±1.8)	13.9 (±1.6)	0.231
Platelet (x10^3^)	238.2 (±54.5)	237.2 (±61.2)	239.7 (±49.2)	0.962
Glucose (mg/dl)	149.5 (±17.5)	154.6 (±21.2)	142.9 (±20.2)	0.513
CK-MB	155.9 (73.5-285.2)	132.2 (61.9-215.5)	147.3 (71.1-256.3)	0.372
Troponin-I (ng/dl)	75.4 (33.3-95.2)	76.3 (38.5-102.3)	77.3 (41.1-101.2)	0.989
LDL-cholesterol (mg/dl)	122.6 (82.5-162.1)	128.8 (77.9-158.2)	123.2 (71.1-160.3)	0.592
Creatinine (mg/dl)	0.97 (±0.18)	0.92 (±0.21)	0.94 (±0.19)	0.658
GFR (ml/dk/1.73 m^2^)	83.1 (±17.4)	84.5 (±16.1)	91.6 (±20.1)	0.364
Symptom-to-balloon time (minutes)	307.3 (189.5-405.2)	288.8 (141.2-355.1)	295.1 (144.5-362.2)	0.893
Door-to-balloon time (minutes)	21.2 (14.8-26.5)	21.5 (12.2-30.1)	21.3 (11.2-28.9)	0.922
Ejection fraction [n (%)]	35.9 (± 8.7)	38.7 (±7.4)	39.8 (±6.6)	0.174
Stent diameter (mm)	3.10 (2.46-3.70)	3.16 (2.55-3.42)	3.09 (2.48-3.41)	0.670
Stent length (mm)	25.09 (15.8-32.2)	25.61 (14.1-30.9)	25.25 (17.8-31.3)	0.966
Clopidogrel [n (%)]	71 (95.8%)	106 (99.1%)	79 (98.8%)	0.137
Acetylsalicylic acid [n (%)]	72 (97.3%)	107 (100.0%)	76 (95.0%)	0.075
Beta-blocker [n (%)]	66 (89.2%)	99 (92.5%)	75 (93.8%)	0.560
RAAS blocker [n (%)]	65 (87.8%)	98 (91.6%)	72 (90.0%)	0.718
Statins [n (%)]	67 (90.5%)	101 (94.4%)	75 (93.8%)	0.581
Gp IIb-IIIa inhibitors [n (%)]	11 (16.2%)	16 (15.8%)	5 (7.0%)	0.178
SYNTAX score	24.2 (±8.9)	25.7 (±7.7)	26.2 (±11.3)	0.311
Intensive care unit (days)	3.20 (±0.9)	2.69 (±1.6)	3.16 (±1.1)	0.314
Total hospital stay (days)	6.00 (±1.9)	5.36 (±1.7)	6.15 (±1.9)	0.245

Data are expressed in numbers (%), mean±1SD, or median and interquartile range.

Percentages are rounded.

* Statistically significant.

BMI=body mass index; CK-MB=creatinine kinase-myocardial band; DBP=diastolic blood pressure; GFR=glomerular filtration rate; Gp=glycoprotein; LDL=low-density lipropotein; RAAS=renin-angiotensin-aldosterone system; SBP=systolic blood pressure; SD=standard deviation

**Fig. 1:**
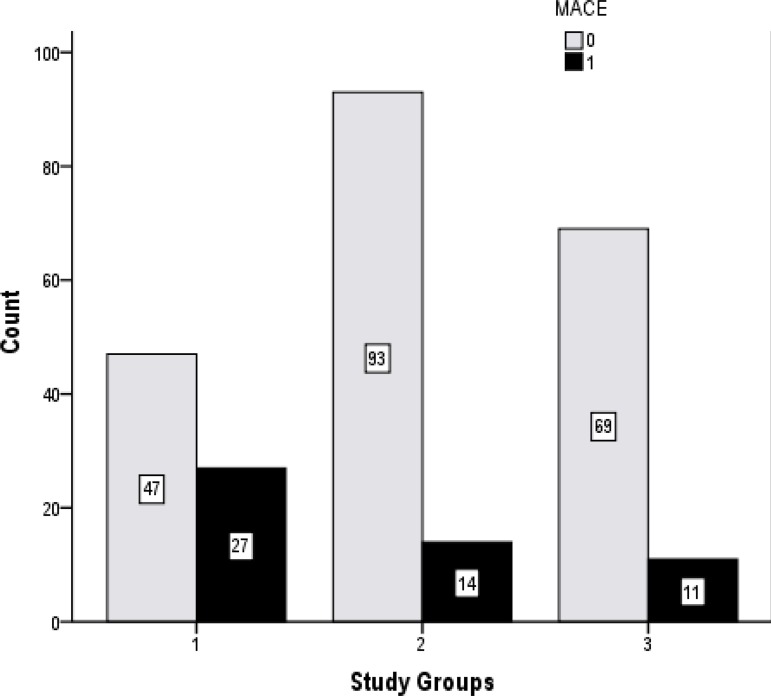
Distribution of patients with major adverse cardiac events (MACE) during one-year follow-up according to the study groups.

Mortality, MACE, cardiogenic shock, and newly-onset atrial fibrillation were more frequent in Group 1 compared to other groups. Re-MI, major stroke, mechanical complication, ventricular tachycardia, major bleeding, and TIMI flow grade <III were similar. The distribution of adverse events, which were seen during the one-year of follow-up, is shown in Table 2 and Figure 1, according to the groups.

**Table 2:** Morbidity, mortality, and MACE rates of in-hospital and post-PPCI one-year period.

Variable	Low PP (n=74)	Moderate PP (n=107)	High PP (n=80)	*P* value
MACE [n (%)]	27 (36.5%)	14 (13.1%)	11 (13.8%)	<0.001*
Mortality in one year [n (%)]	10 (13.5%)	2 (1.9%)	2 (2.5%)	0.001*
Killip class-I [n (%)]	41 (55.4%)	88 (82.2%)	67 (83.8%)	<0.001*
Killip class-II [n (%)]	14 (18.9%)	13 (12.1%)	8 (10.0%)	0.236
Killip class-III [n (%)]	6 (8.1%)	3 (2.8%)	4 (5.0%)	0.314
Cardiogenic shock after PPCI [n (%)]	13 (17.6%)	3 (2.8%)	1 (1.3%)	<0.001*
Re-MI [n (%)]	4 (5.4%)	6 (5.6%)	6 (7.5%)	0.798
Major stroke [n (%)]	2 (2.7%)	2 (1.9%)	2 (2.5%)	0.924
Atrial fibrillation after STEMI [n (%)]	6 (8.1%)	1 (0.9%)	1 (1.3%)	0.012*
Mechanical complication [n (%)]	3 (4.4%)	1 (0.9%)	2 (2.5%)	0.381
Ventricular tachycardia [n (%)]	6 (8.1%)	2 (1.9%)	2 (2.5%)	0.075
Major bleeding [n (%)]	5 (6.8%)	1 (0.9%)	4 (5.0%)	0.108
TIMI-0 flow rate after PPCI [n (%)]	4 (5.4%)	3 (2.8%)	3 (3.8%)	0.675
TIMI-I/II flow rate after PPCI [n (%)]	9 (12.2%)	5 (4.7%)	5 (6.3%)	0.148

Data are expressed in numbers (%), mean±1SD, or median and interquartile range.

Percentages are rounded.

* Statistically significant.

MACE=major adverse cardiac events; MI=myocardial infarction; PP=pulse pressure; PPCI=primary percutaneous coronary intervention; SD=standard deviation; STEMI=ST-elevation myocardial infarction; TIMI=thrombolysis in myocardial infarction

**Fig. 2:**
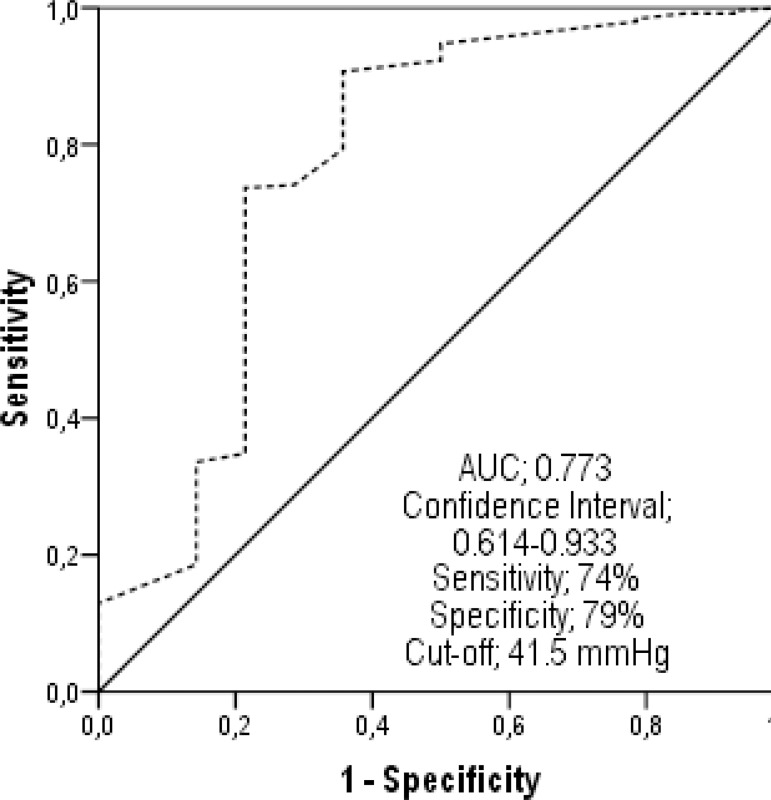
The pulse pressure value which can predict the occurrence of mortality during one-year follow-up was determined as 41.5 mmHg in receiver operating characteristics analysis. AUC=area under the curve

**Fig. 3:**
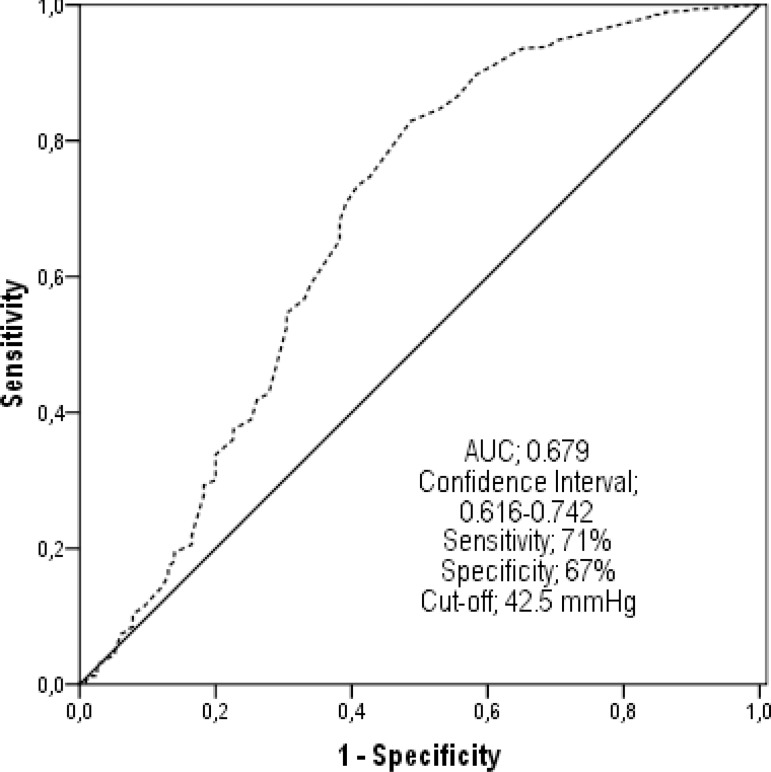
The pulse pressure value which can predict the occurrence of major adverse cardiac events (MACE) rates during one-year follow-up was determined as 42.5 mmHg in receiver operating characteristics analysis. AUC=area under the curve

**Fig. 4:**
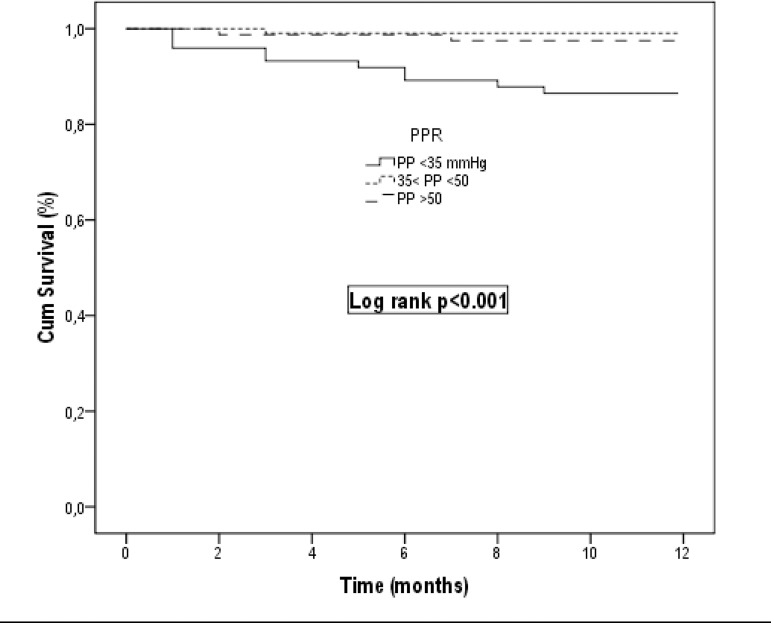
One-year survival rate in Group 1 was detected lower than in other groups in Kaplan-Meier survival analysis. Cum=cumulative; PP=pulse pressure; PPR=pulse pressure ratio

In the ROC analysis, the predictive PP value was calculated as 42.5 mmHg for MACE development and 41.5 mmHg for mortality (Figure 2, Figure 3). The patients' annual life expectancy assessed by the Kaplan-Meier method was lower in Group 1 (Figure 4). There was a low positive correlation between LVEF, PP, and StD.

After univariate and multivariate analyses carried out with the variables that could affect mortality, LVEF, inability to initiate beta-blockers in the acute period, PP, SBP, GFR, and STD were determined to be mortality predictors (Tables 3 and 4). After additional adjustment for SBP, PP remained an independent predictor of mortality (hazard ratio [HR] 2.1 [1.2 to 4.3], *P*=0.034 *vs*. HR 1.6 [1.1 to 3.1], *P*=0.041).

**Table 3:** Variables which effect MACE according to univariate analysis results.

Predictor variables	OR (95% CI)	*P*
Age	0.7 (0.4-1.1)	0.599
Gender (female)	1.4 (0.7-1.9)	0.048
Diabetes mellitus	0.8 (0.5-1.3)	0.344
SBP	1.9 (1.0-3.1)	0.010
DBP	0.9 (0.5-1.5)	0.167
PP	2.5 (1.4-5.1)	0.003
SYNTAX score	0.7 (0.5-1.0)	0.484
Ejection fraction	5.7 (2.8-8.1)	<0.001
No-beta-blocker	3.7 (1.6-7.0)	<0.001
No-RAAS blockers	1.7 (1.0-3.0)	0.022
Symptom-to-balloon time	1.9 (0.9-3.6)	0.004
Door-to-balloon time	1.0 (0.7-1.5)	0.198
Gp IIb-IIIa receptor blockers	0.8 (0.4-1.8)	0.323
Haemoglobin	1.1 (0.7-1.4)	0.144
GFR	1.9 (0.9-2.7)	0.012

CI=confidence interval; DBP=diastolic blood pressure;

GFR=glomerular filtration rate; Gp=glycoprotein;

MACE=major adverse cardiac events; OR=odds ratio;

PP=pulse pressure; RAAS=renin-angiotensin-aldosterone system; SBP=systolic blood pressure

**Table 4:** Variables which effect MACE according to multivariate analysis results.

Predictor variables	OR (95% CI)	*P*
Age	__	__
Gender (female)	0.8 (0.7-1.3)	0.124
Diabetes mellitus	__	__
SBP	1.9 (0.9-2.8)	0.046
DBP	__	__
PP	2.3 (1.6-4.7)	0.032
SYNTAX score	__	__
Ejection fraction	4.1 (1.9-6.9)	<0.001
No-beta-blocker	2.9 (1.6-5.1)	0.014
No-RAAS blockers	1.1 (0.4-2.0)	0.098
Symptom-to-balloon time	2.4 (1.4-3.9)	0.037
Door-to-balloon time	__	__
Gp IIb-IIIa receptor blockers	__	__
Haemoglobin	__	__
GFR	2.1 (0.8-3.2)	0.048

CI=confidence interval; DBP=diastolic blood pressure;

GFR=glomerular filtration rate; Gp=glycoprotein;

MACE=major adverse cardiac events; OR=odds ratio;

PP=pulse pressure; RAAS=renin-angiotensin-aldosterone system; SBP=systolic blood pressure

## DISCUSSION

In our study, it was determined that the PP measured in A-STEMI patients intra-aortically before PPCI was significantly associated with MACE.

Many studies have discussed the relationship between blood pressure and prognosis for many years. The first blood pressure studies suggested that DBP is the main determinant of prognosis^[11]^. The studies in the following years suggested the idea that SBP is more important and it is not necessary to evaluate DBP for prognosis^[12]^. Afterwards, the studies that evaluated the relationship between ACS and blood pressure were concluded. SBP < 90 mmHg after AMI was generally considered to be a poor prognostic indicator^[13]^. Various risk scoring systems were developed as a result of this data. Blood pressure irregularity was rated as a significant negative indicator^[5]^. It is known that high blood pressure in patients with ACS is associated with cardiovascular complications as much as low blood pressure. AMI blood pressure studies usually assessed SBP levels. However, in a small number of studies, it was determined that the DBP not within the normal limits after ACS was associated with increase in mortality and morbidity^[14]^. All these evaluations showed that blood pressure out of the normal limits in AMI patients is a bad indicator. The changes in PP calculated according to SBP and DBP are indicative of stiffness of vascular bed. The dramatic decrease in CO and peripheral resistance in the patients with ACS, heart failure, aortic stenosis, and septic shock were shown to cause a decrease in PP. Significant changes in PP were determined to increase morbidity and mortality^[15]^.

Li et al.^[7]^ indicated that PP lower than 50 mm and higher than 70 mmHg increased the mortality rates significantly in patients with ACS who were over 80 years old. Patients with PP between these two values had the best prognosis. In this study, no upper or lower limit was determined for blood pressure values. Patients with blood pressure values between 170/100 mmHg and 130/60 mmHg were considered similarly since their PP was 70 mmHg^[7]^. In our study, A-STEMI patients with normal SBP and DBP were followed according to the recommendations of current guidelines. Thereby, the association of PP with the emerging cardiovascular events in patients with normal SBP and DBP intervals was attempted to be determined. Patients in Group 1 who had PP lower than 35 mmHg were found to have the worst prognosis. The highest mortality and MACE rates were also in this group. Li et al.^[7]^ e El-Menyar et al.^[8]^ also showed that PP reduction in patients with ACS was associated with stroke and mortality. In both studies, PP was calculated according to the blood pressure values measured from the arm by experienced health personnel. Li et al.^[7]^ followed-up the patients with AMI while El-Menyar et al.^[8]^ followed-up all patients with ACS. As it is known, in all the ACS subgroups, CO does not fall to the same level, and the neuroendocrine system is not activated at the same degree. Additionally, cardiac functions do not change at similar levels in all STEMIs. Blood pressure levels tend to be lower due to decreased stroke volume and increased vagal stimulation in inferior STEMI^[16,17]^. Therefore, only A-STEMI patients were accepted in order to create a homogenous group.

In the previous studies^[9,10]^, the central blood pressure obtained with the aid of aortic catheter was shown to have a stronger association with cardiovascular outcomes than that measured from the arm. Thus, central blood pressure measurements obtained from the ascending aorta with the aid of catheter before PPCI in the coronary angiography laboratory were taken into consideration in our study. As a result of one-year follow-up, an increased number of deaths, cardiogenic shocks after PPCI, and total MACE ratios were detected in Group 1 compared to other groups. The prevalence of LVEF and coronary artery disease was similar among the groups. The rate of female patients in Group 1 was higher than in other groups. As it is known, the pain threshold is higher in women, and the microvascular circulation dynamics and complication rates after AMI are increased compared to men^[18,19]^. These findings are supported by the high number of female patients in Group 1 with higher rates of mortality and MACE in our study.

When the Kaplan-Meier curves were evaluated, the survival rate in Group 1 was worse than in other groups. There was no difference in survival expectancy between Group 2 and Group 3. The PP level limit, which could increase MACE rates, was determined as 42.5 mmHg in the ROC analysis. The PP value, which could predict one-year mortality, was found to be 41.5 mmHg. The risk of developing cardiovascular events was determined to be increased below these values. According to multivariate analysis, the predictors of mortality were determined to be PP, SBP, lower LVEF, inability to initiate beta-blockers in the acute phase, lower GFR, and STD time.

Statistical analyses revealed that SBP and PP were associated with the complications that could develop. However, the predictive power of PP was found to be higher than the one of SBP (Table 3). This may be due to the fact that patients with SBP values within the normal range were included. Also, PP is directly related to cardiac perfusion^[20-22]^. Myocardial perfusion pressure decreases with the decrease of PP. Even if SBP is decreased, the PP can maintain coronary perfusion by staying constant for a certain period. However, the reduction of PP reduces coronary perfusion. Myocardium recovery may be delayed after A-STEMI and sometimes may not be possible due to decreased perfusion. The reduction in PP is an important condition which can increase cardiovascular complication rates due to these reasons.

In our study, the inability to initiate beta-blockers in the acute phase of the disease was one of the poor prognostic factors. The patients in whom beta-blockers could not be initiated included those who generally had hemodynamic instability and were unable to optimise medical treatments fully. Therefore, we think that there is a significant relationship between beta-blockers and prognosis.

Li et al.^[7]^ showed that there is a positive correlation between LVEF and PP. There was no significant difference between the groups in terms of the mean LVEF in our study. However, in the correlation analyses, it was determined that there was a low positive correlation between the decline in PP and LVEF and the prolongation of StD period. This condition suggests that cardiovascular complications following STEMI are associated with other variables apart from LVEF. It may be useful to consider the changes in PP other than LVEF.

### Limitations

Our study's main limitation was the low number of patients. The study was conducted in one centre. Blood pressure values are dynamic parameters especially in patients with STEMI, and sedation, agitation, and inotropic agents may affect these values. In our study, patients with hemodynamically unstable requirement of inotropic agents and IABP were excluded. We can't extend this result's validity to hypotensive patients due to the fact that hemodynamically stable patients were included in our study. The normalisation of PP with systolic arterial pressure (PAS) or diastolic arterial pressure (PAD) might be beneficial to predict the adequacy of coronary perfusion. PP has been registered with a water-filled catheter and not with high-fidelity catheters. This may reduce the quality of the measurement in patients with lower PP. Although poorly significant differences emerged between Group 2 and Group 3, patients were divided into three groups to see the effect of the extreme values on the results. Some of the recommended pharmacological treatments after STEMI in the current guidelines could not be initiated in the patients with hemodynamic instability at the same or in similar doses to the other patients. The left ventricular end-diastolic pressure (LVEDP) could not be calculated by entering the left ventricle to avoid time loss since the patients had A-STEMI. If LVEDP could have been calculated, more accurate results could have been obtained by assessing perfusion pressure. These measurements can be performed in STEMI patients whose general conditions are stable. In our study, there was a significant difference between the groups in terms of means of SBP. Although SBP values were not the same, unlike other studies, they were in the range considered as normal by guidelines.

## CONCLUSION

In our study, the central PP measured intra-aortically before PPCI in patients with A-STEMI in the angiography laboratory was significantly associated with MACE. Therefore, even if the blood pressure of patients with A-STEMI is within normal limits, it is good to evaluate the changes in PP carefully while arranging follow-up and treatment. Including PP in the algorithms while calculating the risk levels of patients after AMI might be beneficial.

**Table:** 

**Authors’ roles & responsibilities**	
IG	Analysis and interpretation of data; drafting the paper; revising the work; approval of the final version
LC	Analysis and interpretation of data; drafting the paper; revising the work; approval of the final version
BS	Conception and design of the work; acquisition of data; analysis and interpretation of data; drafting the paper; revising the work; approval of the final version
MBA	Conception and design of the work; acquisition of data; analysis and interpretation of data; drafting the paper; revising the work; approval of the final version
HK	Conception and design of the work; acquisition of data; analysis and interpretation of data; drafting the paper; revising the work; approval of the final version
ZC	Conception and design of the work; acquisition of data; analysis and interpretation of data; drafting the paper; revising the work; approval of the final version
BY	Conception and design of the work; acquisition of data; analysis and interpretation of data; drafting the paper; revising the work; approval of the final version
SU	Conception and design of the work; acquisition of data; analysis and interpretation of data; drafting the paper; revising the work; approval of the final version
HD	Conception and design of the work; acquisition of data; analysis and interpretation of data; drafting the paper; revising the work; approval of the final version
